# Clinical and epidemiological profiles including meteorological factors of low respiratory tract infection due to human rhinovirus in hospitalized children

**DOI:** 10.1186/s13052-017-0346-z

**Published:** 2017-03-07

**Authors:** Yongdong Yan, Li Huang, Meijuan Wang, Yuqing Wang, Wei Ji, Canhong Zhu, Zhengrong Chen

**Affiliations:** Department of Respiratory Disease, Children’s Hospital of Soochow University, NO.303, Jingde Road, Suzhou, Jiangsu Province 215003 People’s Republic of China

**Keywords:** Human Rhinovirus, Meteorological factors, Lower respiratory tract infection, Children, Epidemiology

## Abstract

**Background:**

Lower respiratory tract infection (LRTI) is a major cause of morbidity and mortality in children. Human rhinovirus (HRV) is confirmed to be associated with pediatric lower respiratory tract infection. Seasonal and meteorological factors may play a key role in the epidemiology of HRV. The purposes of this study were to investigate the frequency, seasonal distribution, and clinical characteristics of hospitalized children with LRTI caused by HRVs. In addition, associations between incidence of HRVs and meteorological factors in a subtropical region of China were discussed.

**Methods:**

Hospitalized children <14 years old admitted to the Respiratory Department of the Children’s Hospital, which is affiliated to Soochow University, between January 1, 2013 and December 31, 2015, were enrolled in this study. Multi-pathogens were detected in nasopharyngeal aspirate samples. Meanwhile, meteorological factors were recorded.

**Results:**

The average incidence of HRVs infection was 11.4% (707/6194) and 240 cases of which were co-infection cases with other pathogens. Children with co-infection presented more frequent fever and tachypnea compared to children infected with HRVs only (both *P* < 0.05). Among 707 HRV positive children, the mean age was 23.2 months (range 1 to 140 months). Among all respiratory infections, the highest incidence of HRVs cases occurred in children age 13–36 months old (15.1%, 203/1341). Of all 228 HRV cases in 2014, 85 cases (37.3%) were HRV-C positive. HRVs and HRV-C infection occurred throughout the year during the study period, although a higher incidence was observed in summer and autumn seasons. HRVs or HRV-C incidence in hospitalized children with LRTI was associated with the monthly mean temperature (both *P* < 0.05).

**Conclusion:**

HRV was one of the most common viral pathogen detected in hospitalized children with LRTI at the Children’s Hospital of Suzhou, China, and had its own seasonal distribution including HRV-C, which was partly caused by temperature.

## Background

Viruses are the most frequent cause of acute respiratory infections and are a leading cause of childhood mortality [1]. However, the etiological agents remain unknown in a significant proportion of cases. Human rhinovirus (HRV), first discovered in the 1950’s, is a member of the family *Picornaviridae*, genus *Enterovirus*, and is one of the most frequent cause of acute respiratory infections. Recent studies showed that HRV not only causes mild upper respiratory tract infections but also associated with lower respiratory tract infections (LRTIs) [2], acute asthma exacerbations [3], recurring wheeze [4] and severe cases with bronchiolitis [5] particularly in infants and early childhood [6].

Global HRVs activity varies substantially from year to year among different populations and regions and recent epidemiological data show that HRVs are present all year round with different incidence rates from 16–68.5% in children with LRTI [2, 7–9]. HRVs are the most common cause of respiratory viral illness during the spring, summer, and fall seasons, while respiratory syncytial virus (RSV) and influenza virus (IV) predominate in the winter [10, 11]. However, no seasonal pattern of HRVs was found in Istanbul, Turkey [12]. In general, seasonal distribution of HRVs varies in different regions and is still poorly understood. Recent studies show that meteorological factors have the greatest potential to play key roles in epidemics and seasonality of respiratory virus infection [13–15]. Among these meteorological factors, temperature and humidity are frequently associated with respiratory virus infection. However, few studies investigated the association between HRVs activity and meteorological factors in subtropical area of China.

The objective of this study was to ascertain the frequency, seasonal and clinical characteristics of HRVs in hospitalized children with LRTI and evaluate the effects of meteorological factors on the incidence of HRV in a subtropical area in China.

## Methods

### Study design

From January 2013 to December 2015, children < 14 years old with LRTIs admitted to Department of Respiratory Disease in Children’s Hospital of Soochow University were prospectively enrolled in this study. After completing a full clinical record, nasopharyngeal aspirate (NPA) was performed on each child enrolled as described previously [16] and stored at 4 °C until sent to the clinical laboratory for further direct immunofluorescence assay and polymerase chain reactions (PCR) analysis. Pending the molecular diagnostic, all samples were stored at −80 °C after DNA or RNA extraction. LRTI (bronchiolitis, bronchitis, pneumonia and asthma exacerbation) was defined by the presence of signs and symptoms of an acute respiratory infection (cough, nasal discharge, with or without fever), lower respiratory signs (tachypnea, retractions, prolonged expiratory time, or crackles/wheezing on auscultation) and radiologic evidence of LRTI. Chest radiography was performed using standard equipment and radiographic techniques, and reviewed by the radiologists in digital format. Generally speaking, bronchiolitis presents bilateral hyperinflation with variable, patchy areas of atelectasis. Pneumonia presents increased shadowing or consolidation while increased lung markings shows in bronchitis or asthma exacerbation. Children with a history of chronic lung disease, underlying immunodeficiency states, or preexisting cardiac, renal, neurologic, or hepatic dysfunction, or bronchopulmonary malformation were excluded from the study. This study was conducted with the approval of the Institutional Human Ethical Committee of Children’s Hospital of Soochow University. An informed consent was obtained from all the subjects or guardians who participated in this study.

### Detection of HRVs and HRV-C using real time PCRs

In present study, a sensitive quantitative PCR method targeted a 210-bp region from the conserved 5’ untranslated region (5’ UTR) was used to screen for HRV modified from a previous study [17]. The primers and probe sequences were HRV-F: 5’-TGGACAGGGTGTGAAGAGC -3’;HRV-R:5’-CAAAGTAGTCGGTCCCATCC-3’ ; HRV-probe: FAM-TCCTCCGGCCCCTGA ATG-TAMRA. Nucleic acids were extracted from NPA samples with TRIzol reagent (Life Technologies, Carlsbad, USA) following the protocol provided by the manufacturer. A final 200 μl of RNA was eluted and reverse transcription reactions were performed with M-MLV reverse transcriptase (Promega, Madison, USA) and random hexamers (Sangon Biotech, Shanghai, China) for cDNA synthesized at 37 °C for 60 min according to the manufacturer’s specifications. PCRs were performed in a volume of 25 μl containing 3 μl of sample DNA, 0.25 μl of 5 U/μl TaqMan (Promega, Madison, USA), 75% DEPC treated water 14.75 μl, buffer solution 2.5 μl, 25 mM MgSO_4_ 2 μl, 10 mM dNTP 1 μl, forward, reverse primers (1.5 μmol/L) and probe (0.2 μmol/L) each 0.5 μl. The PCR conditions were as follows: reverse transcription, 30 min at 50 °C; polymerase activation, 15 min at 95 °C; and 45 cycles of 30 s at 95 °C and 60 s at 60 °C. The concentration of each detected sample was then calculated automatically according to standard curve.

From January 2014 to December 2014, positive HRV samples were used to determine HRV-C strain. According to analysis of HRV VP4/VP2 coding region sequences from GenBank, the conserved sequence (8–37 amino acid) from VP2 was confirmed to differentiate HRV-C from HRV-A and -B after comparison of 52 newly identified HRV-C subtypes using a phylogenetic tree. The degenerate primers were designed according to the conserved sequence. The degenerate primers sequences were HRV-F: 5’-GGNWWBTCYGATAGGCTHAA-3’;HRV-R:5’-TANBBDGGCCAYTCHCCRTANGC-3’(N = A,T, C or G, W = A or T, B = G, T or C, Y = C or T, H = A, T or C, D = G, A or T, R = A or G,V = G, A or C). Compared to positive control sample of HRV-51 strain, the optimal concentration of primers and melting temperature were obtained. Then all the screened HRV positive samples were amplified by real time PCR and HRV-C subtypes could be confirmed by comparing to high-resolution melting curve of HRV-C51.

### Detection of other common viruses and *Mycoplasma pneumoniae* (MP)

Samples were equally analyzed for seven common viruses, including RSV, IV-A and IV-B, parainfluenza viruses 1, 2, 3 (PIV-1, 2, 3), and adenovirus (ADV) using direct immunofluorescence (DFA) with virus-specific fluorescence-labeled monoclonal antibodies (Diagnostic Hybrids, Athens, USA) and ultraviolet light microscopy. Human metapneumovirus (hMPV) were detected by reverse transcription PCR whereas human bocavirus (HBoV) and Mycoplasma pneumonia (MP) were detected by real time PCR respectively as previously described [14]. Briefly, for hMPV detection, primers were designed to specifically amplify the N gene (213 bps). The forward and reverse primers were hMPV-F: 5’-AACCGTGTACTAAGTGATGCACTC-3’ and hMPV-R: 5’-CATTGTTTGACCGGCCCCATAA-3’, respectively. For HBoV VP1 gene detection, the primers and probe sequences were HBoV-F: 5’-TGACATTCAACTACCAACAACCTG-3’;HBoV-R:5’-CAGATCCTTTTCCTCCTCCAATAC-3’ ;HBoV-probe: FAM-AGCACCACAAAACACCTCAGGGG-TAMRA. As for MP detection, another fluorescent real-time PCR was performed to identify the 16S rRNA gene of MP. The primers and probe sequences were MP-F: 5’-GCAAGGGTTCGTTATTTG-3’ and MP-R: 5’-CGCCTGCGCTTGCTTTAC-3’, and MP-probe: FAM-AGGTAATGGCTAGAGTTTGACTG-TAMRA. MP and HBoV positive samples were defined with a concentration of DNA >2.5 × 10^3^copies/ml to exclude the MP or HBoV colonization.

### Meteorological data collection

Meteorological data for Suzhou, including daily mean temperature (°C), mean relative humidity (%), total month rainfall (mm), sum of sunshine (h), and mean wind velocity (m/s), were obtained from Suzhou Weather Bureau at longitude 120°6’ east and latitude 31°3’ north, which is located 8 km away from the hospital. Meteorological data were obtained hourly, and average daily values were calculated. Monthly means were calculated using the daily means for temperature, relative humidity, and wind velocity. Total rainfall and hours of sunshine were calculated as a total measurement for the month.

### Statistical analysis

The continuous variables were compared using the Student t test or Mann–Whitney *U* test if the data were abnormal in distribution. Categorical data were analyzed using the Mentel-Haenszel or chi-squared (*χ2*). Correlations of incidence of HRV with meteorological factors were evaluated using Pearson’s or Spearman rank correlation. Because of colinearity between meteorological factors, associations between meteorological factors and HRV incidence were also analyzed using Linear Regression. *P* < 0.05 was considered to be statistically significant.

## Results

### Viral and atypical bacterial etiology of LRTIs

A total of 6196 NPA samples were available and 276 children were not enrolled because of a refusal to participate from their parent or presenting underlying disease. Of 6196 NPA samples, 3882 (62.7%) were positive for at least one pathogen and the most commonly identified pathogen was *Mycoplasma pneumonia* (MP) (22.1%, 1369/6196), followed by RSV (12.0%, 744/6196), HRV (11.4%, 707/6196), human bocavirus (HBoV) (5.7%, 354/6196), PIV-3 (5.1%, 315/6196), human metapneumovirus (hMPV) (3.7%, 230/6196), adenovirus (ADV) (1.1%, 69/6196), parainfluenza virus 1 (PIV-1) (0.9%, 55/6196), IV-A (0.5%, 32/6196), and parainfluenza virus 2 (PIV-2) (0.1%, 7/6196). Among 707 HRV-positive cases, 467 (66.1%) were infected only with HRV and 240 (33.9%) were co-infected with other pathogens. The most commonly co-detected pathogen with HRV was *Mycoplasma pneumoniae*, followed by HBoV and RSV (Table 1). Of all the 228 HRVs cases in 2014, HRV-C was detected in 85 cases and accounting for 37.3%.

**Table 1:** Frequency of HRV infection with other respiratory pathogens in children with LRTIs

Pathogen distribution of co-infections with HRV	Cases	%
*Mycoplasma pneumoniae*	121	50.4
HBoV	47	19.6
RSV	27	11.3
PIV-3	17	7.1
ADV	7	2.9
hMPV	7	2.9
More than two pathogens	14	5.8
Total co-infections	240	100

*LRTI* lower respiratory tract infection, *HRV* human rhinovirus, *HBoV* human bocavirus, *RSV* respiratory syncytial virus, *hMPV* human metapneumovirus, *PIV* parainfluenza virus, *ADV* adenovirus

### Demographic and clinical characteristics of children infected by HRV with or without co-infection

**Fig. 1:**
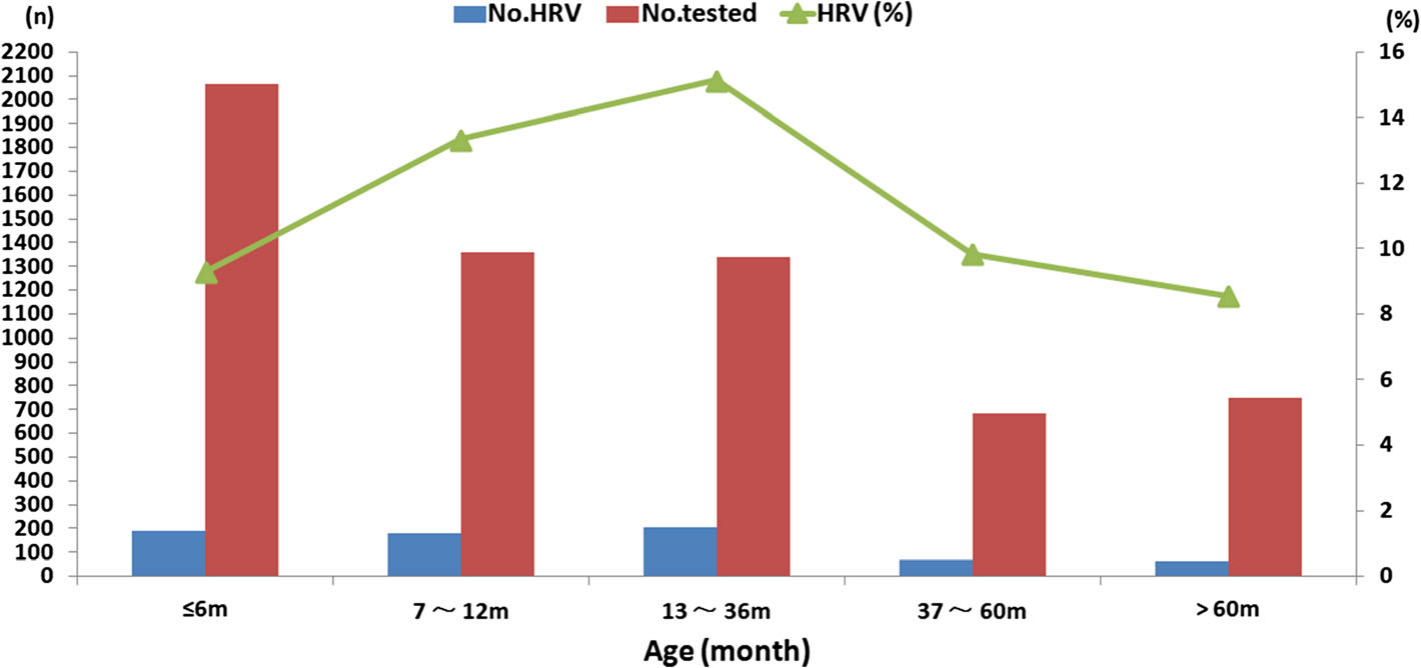
Age distribution in hospitalized children with lower respiratory tract infection due to human rhinovirus (HRVs) infection

Of the 707 HRV positive cases, the mean age was 23.2 months (range 1 to 140 months) and the ratio of male to female was 2.3:1 and slightly higher than 1.7:1 of non-HRV infected cases (*P* = 0.052). HRV affected mostly children aged 13–36 months with an incidence of 15.1% (203/1341). The HRV incidence in different age groups was as follows: 1–6 months (9.3%, 192/2065), 7–12 months (13.3%, 181/1357, 13–36 months (15.1%, 203/1341), 37–60 months (9.8%, 67/682), and > 60 months (8.5%, 64/749) and HRV incidence was significantly different between groups (*χ2 =* 40.31, *P* < 0.0001) as shown in Fig. 1. The demographic and clinical characteristics of cases with single HRV infection and co-infection are summarized and compared in Table 2. Children with co-infection presented more frequent fever, tachypnea and longer hospital stay compared to children infected with HRVs only (all *P* < 0.05). However, there was no difference of clinical characteristics between HRV-C infection and HRV-A/B infection (data not shown).

**Table 2:** Demographic and clinical characteristics of children infected by HRVs with or without co-infection

Parameters	Single infection	Co-infections	*P* Value
Demographic characteristics			
Age, mean (25–75%)	23.9 (5.8–29)	22.0 (5.9–31)	0.988
Sex, male (%)	310 (66.4)	162 (67.5)	0.765
Median duration of stay in hospital, days	7.6 ± 2.7	8.4 ± 2.7	0.017
Clinical manifestation			
Cough, n (%)	452 (96.8)	232 (96.7)	0.931
Wheezing, n (%)	225 (48.2)	118 (49.2)	0.804
Rhinorrhea, n (%)	206 (44.1)	91 (37.9)	0.114
Fever, n (%)	186 (39.8)	125 (52.1)	0.002
Tachypnea, n (%)	105 (22.5)	74 (30.8)	0.016
Dyspnea, n (%)	52 (11.1)	27 (11.3)	0.963
Cyanosis, n (%)	10 (2.1)	5 (2.1)	0.960
Laboratory examination			
White blood cells, (×10^9^/ml)	11.7 ± 5.0	11.1 ± 4.3	0.380
Neutrophils, (%)	43.7 ± 21.5	47.9 ± 20.4	0.173
Platelets, (×10^9^/ml)	380 ± 121	382 ± 115	0.920
C-reaction protein, mg/L, mean (25%–75%)	5.4 (0.1–8.4)	6.3 (0.1–11.8)	0.380

### Seasonal distribution of HRV infection and association with meteorological factors

**Fig. 2:**
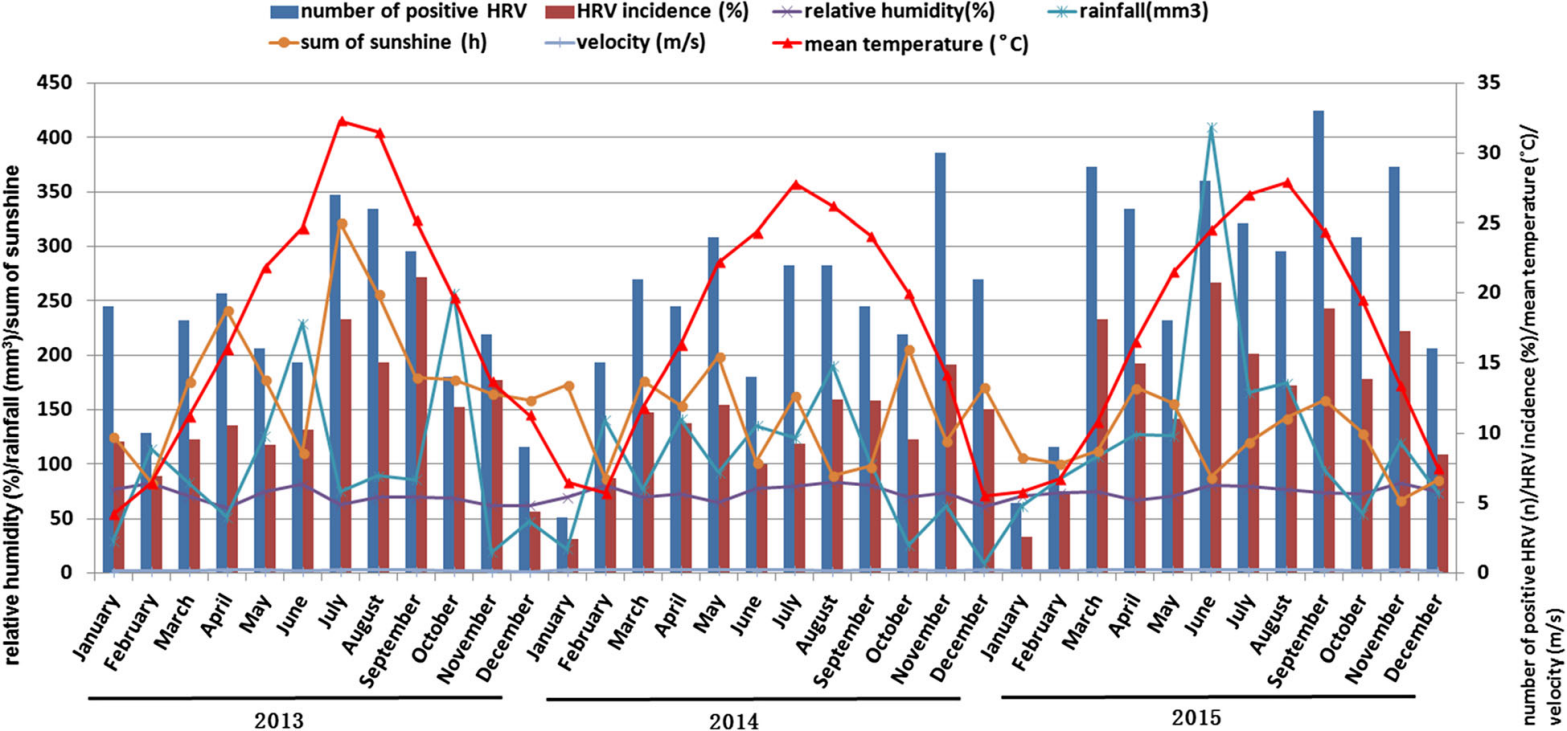
Seasonal and monthly distribution of human rhinovirus (HRVs) infection and meteorological factors from January 2013 to December 2015

**Fig. 3:**
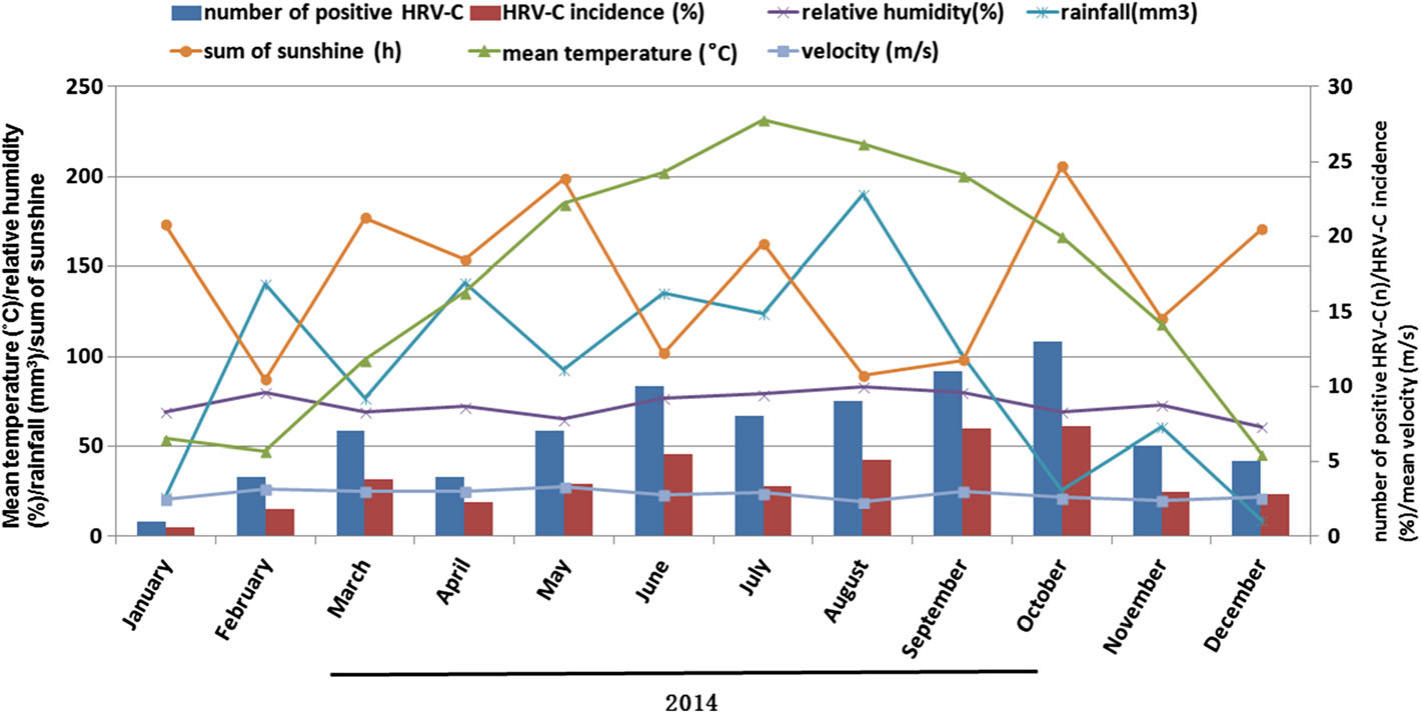
Seasonal and monthly distribution of HRV-C infection and meteorological factors from January 2014 to December 2014

As shown in Fig. 2, HRVs infection occurred throughout the year during the study period, although a higher incidence was observed in summer and autumn seasons, especially in June 2015 (20.7%, 28/135). The HRV incidence was 14.7% (206/1400) in autumn, followed by 13.3% (203/1532) in summer, 11.8% (190/1612) in spring, and 6.5% (108/1652) in winter. The similar seasonality of HRV-C was shown in Fig. 3. The Suzhou area has a subtropical climate. The monthly mean temperature was 18.2 ± 9.2 (mean ± standard deviation) °C, relative humidity was 69.7 ± 7.4%, total rainfall was 100.5 ± 73.9 mm, sum of sunshine was 181.1 ± 65.8 h, and wind velocity was 1.8 ± 0.5 m/s. The monthly mean data for these meteorological variables over the course of this study are shown in Fig. 2. The associations of total HRVs, HRVs with or without coinfection or HRV-C activity with meteorological factors were performed using Spearman correlations and multivariate regression analysis. Total HRVs, HRVs without coinfection and HRV-C incidence was associated with mean temperature (all *P* < 0.05). No correlation was found between total HRVs, HRVs without coinfection, HRV-C incidence and other meteorological factors by multivariate regression analysis (Table 3). The incidence of HRVs with coinfection was not associated with all meteorological factors (all *P* > 0.05).

**Table 3:** Associations of HRVs incidence and meteorological factors

	total HRVs				HRVs without coinfection				HRV-C			
Climate factors	correlation		Multivariate regression		correlation		Multivariate regression		correlation		Multivariate regression	
	r_s_	*P* value	β	*P* value	r_s_	*P* value	β	*P* value	r_s_	*P* value	β	*P* value
Temperature (°C)	0.545	0.001	0.304	0.001	0.648	<0.001	0.709	0.002	0.660	0.019	1.002	0.032
Relative humidity (%)	0.041	0.811	−0.028	0.850	0.030	0.860	−0.262	0.326	0.268	0.399	−0.041	0.946
Total rainfall (mm)	0.322	0.056	0.113	0.482	0.360	0.031	0.173	0.345	0.109	0.736	−0.747	0.176
Sum of sunshine (h)	0.166	0.332	−0.039	0.804	0.212	0.215	−0.174	0.549	−0.111	0.731	−0.503	0.368
Wind velocity (m/s)	0.168	0.328	−0.012	0.940	0.106	0.537	−0.111	0.454	−0.067	0.837	0.130	0.667

*P* < 0.05 indicates significant difference

*HRV* human rhinovirus

## Discussion

The findings in this study confirmed the hypothesis that HRVs are common pathogen in hospitalized children with lower respiratory tract infection. To our knowledge, this is the first time to explore the associations between HRVs incidence and meteorological factors in a subtropical region of China. In the present study, HRVs was detected in 11.4% NAP samples collected from January 2013 to December 2015 and this rate is similar to previous report in Lanzhou, China (13.1%, 53/406) [18]. However, the incidence of this study is lower than previous studies in Turkey (18.4%), Vietnam (24.2%) and Thailand (30%) [12, 19, 20]. All the studies above show that HRV plays an important role in respiratory tract infections worldwide.

In the present study, 81.3% (575/707) of total HRVs occured in children younger than 36 months, especially in children 13 to 36 months old which is consistent with recent report in Changsha, China [21]. Meanwhile, Linder et al. [22] reported that HRVs is more frequently detected in younger children and infants than in older children. The possible reason is that younger children are more prone to be sick than older children due to their developing immune system and that parents are more likely to take their younger children to the doctor if they are sick. Moreover, only children hospitalized with LRTIs were included in the study. HRV has been considered to be a benign virus causing mild upper respiratory tract infections, but there is evidence that HRV is also involved in LRTIs, more specifically in bronchiolitis [23] and pneumonia [24], which is similar to our study. In addition, infants hospitalized with HRV bronchiolitis have a great risk of asthma later in childhood [25] and HRV infection is associated with exacerbations of asthma [26]. A recent study presumed that HRV-stimulated peripheral blood mononuclear cells increased the gene expression of ORMDL3 and of GSDMB which was associated with 17q21 variants and correlated with development of asthma [27]. Furthermore, a previous study reported that HRV-C causes more severe respiratory illness in children than HRV-A or –B [28, 29]. In present study, no significant difference of clinical characteristics was found between HRV-C and HRV A/B infection. However, we found that children with co-infection were more severe compared to children infected with HRV only.

Consistent with our study, HRV is the most common cause of respiratory viral illness during the spring, summer, and fall seasons, while RSV predominates in the winter [30]. Factors such as meteorological factors may have an impact in survival and contribute to the seasonal outbreaks of respiratory viruses [13]. Therefore, the recent discussions over the impacts of global warming on the terrestrial environment have awoken the interest of the association between meteorological factors and respiratory tract infections. In previous studies, high relative humidity had been proved to be associated with increasing survival of rhinovirus [13, 31] and it was associated with greater amounts of sick leave in workspaces using humidifiers [32]. However, mean temperature other than relative humidity was positively associated with HRVs or HRV-C incidence in present study. Further studies are necessary to determine why various meteorological factors are associated with HRVs infections in different geographic locations.

Our study has several limitations. First of all, 3-year period of this present study was shorter than other long-term investigations which could cause bias of the HRVs prevalence. Secondly, the data analysis alone may not serve as a conclusive interpretation, since any of associations with meteorological factors may be an indication of other social or environmental factors that also vary with the seasons. In addition, the present study was based on a single center. Taken together, these limitations might have potential biases on the research results.

## Conclusions

HRV was one of the most common pathogen found in hospitalized children aged less than 3 years old with LRTI at the Children’s Hospital of Soochow University, located in a subtropical region of China. The mean temperature of the month was shown to be associated with the seasonal distribution of HRV infection in these cases.

## Abbreviations

ADV: Adenovirus
DFA: Direct immunofluorescence
HBoV: Human bocavirus
hMPV: Human metapneumovirus
HRV: Human rhinovirus
IV: Influenza virus
LRTI: Lower respiratory tract infection
MP: Mycoplasma pneumonia
NAP: Nasopharyngeal aspirate
PCR: Polymerase chain reactions
PIV: Parainfluenza virus
RSV: Respiratory syncytial virus
